# Gypenoside XLIX Ameliorate High-Fat Diet-Induced Atherosclerosis via Regulating Intestinal Microbiota, Alleviating Inflammatory Response and Restraining Oxidative Stress in ApoE^−/−^ Mice

**DOI:** 10.3390/ph15091056

**Published:** 2022-08-26

**Authors:** Ming Gao, Xing Heng, Jing Jin, Weihua Chu

**Affiliations:** 1 State Key Laboratory of Natural Medicines, School of Life Science and Technology, China Pharmaceutical University, Nanjing 210009, China; 2 Nanjing Zhiyi Biotechnology Co., Ltd., Nanjing 210014, China; 3 The People’s Hospital of Lishui, The Sixth Affiliated Hospital of Wenzhou Medical University, The First Affiliated Hospital of Lishui University, Lishui 323050, China

**Keywords:** atherosclerosis, gypenoside XLIX, gut microbiota, inflammation, metabolism

## Abstract

A high-fat choline diet (HFCD)-induced atherosclerosis model in ApoE^−/−^ mice was established to explore the anti-atherosclerotic effects of gypenoside XLIX (GPE). It was found that HFCD-induced atherosclerotic index such as dyslipidemia, atherosclerotic plaque, inflammation, and gut microbiota dysfunction could be reduced by GPE treatment. GPE treatment could decrease Verrucomicrobia, Proteobacteria, and Actinobacteria abundance, and increase Firmicutes and Bacteroidetes population. Moreover, the Firmicutes/Bacteroidetes ratio increased significantly after treatment with GPE. After treatment with GPE, the relative abundance of trimethylamine-producing intestinal bacteria *Clostridioides* and *Desulfovibrionaceae* decreased while butyrate-producing bacteria such as *Eubacterium*, *Roseburia*, *Bifidobacterium*, *Lactobacillus*, *and Prevotella* increased significantly. The GPE group demonstrated higher SCFAs concentrations in the fecal sample, such as Acetic Acid, Propionic Acid, and Butyric Acid. Further pathway analysis showed that 29 metabolic pathways were appreciably disturbed during GPE treatment, including citrate cycle (TCA cycle); galactose and glycero-lipid-metabolism biosynthesis of unsaturated fatty acids, fatty acid biosynthesis. This study suggests that the anti-atherosclerotic effect of GPE is related to the substantial changes in intestinal microbiota and anti-inflammatory activity.

## 1. Introduction

Cardiovascular disease (CVD) is a prevalent disease which has a high mortality rate worldwide [1]. According to the WHO, cardiovascular disease includes a range of diseases which affect the health of blood vessels and heart, including angina pectoris, coronary heart disease, myocardial infarction, hypertension, congenital heart disease, heart valve disease stroke, and cardiomyopathy [2]. Atherosclerosis is one of the most common of these diseases. The feature of atherosclerosis is slow-progressing inflammation in conductance and resistance arteries, thereby causing cholesterol containing low density lipoprotein (LDL) particles which accumulate on the vascular endothelium [3]. Although the cost of medical systems in the world is rising, atherosclerosis is increasing worldwide without any available cure [4]. Atherosclerosis is influenced by multiple factors, and many studies have shown that the gut microbiota is also involved in the occurrence and development of atherosclerosis.. Microbes in the gut form a vast and complex community of interacting organisms, outnumbering the total number of human cells [5]. Recent studies have shown that the gut microbiota contributes to the development of metabolic diseases, particularly to the target of cardiovascular diseases [6]. For example, the relative abundance of butyrate producing bacteria in the gut is significantly and negatively correlated with atherosclerosis [7]. Therefore, improving atherosclerosis by influencing the organismal pathways has drawn widespread attention [8]. There is increasing evidence that various phytochemicals can probably be protective by influencing and remodeling intestinal bacteria [9].

**Figure 1:**
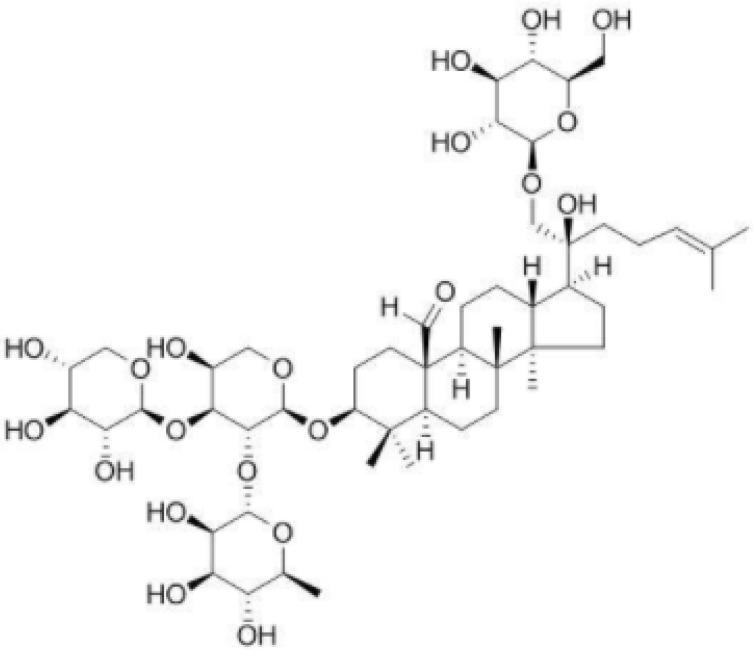
The chemical structure of gypenosides XLIX.

Traditional Chinese herbs are widely used in the clinical treatment of atherosclerosis, such as *Alisma orientale* (Samuel.) Juz, *Salvia miltiorrhiza* Bunge, *Poria cocos* (Schw.) Wolf, and *Chuanxiong Rhizoma*. *Gynostemma* pentaphyllum (Thunb.) Makino is an ancient Chinese herbal medicine and tea. It has effects of lowering blood pressure, blood fat and blood sugar and delaying aging. The main chemical component of *Gynostemma* pentaphyllum (Thunb.) Makino is gypenoside XLIX (GPE) (Figure 1). Gypenosides possessed many pharmacological activities such as anti-cancer, anti-atherogenic and neuroprotection effects, lipid-regulating effects, hepatoprotective effects, and anti-inflammation effects [10,11]. Here we hypothesized that GPE can ameliorate atherosclerosis by regulating gut microbiota and alleviating inflammation.

Apolipoproteins are the major component of circulating high-density lipoprotein (HDL) that inhibits the progression of atherosclerosis [12]. Apolipoprotein E knockout (ApoE^−/−^) mice is a classic model for atherosclerosis research [13]. The aim of this study is to explore the anti-atherosclerosis effect of GPE on the inflammation and intestinal microbiota in a high-fat diet and atherosclerosis induced by choline in ApoE^−/−^ mice.

## 2. Results

### 2.1. Body Weight, Food Intake, and Organ Weight

**Figure 2:**
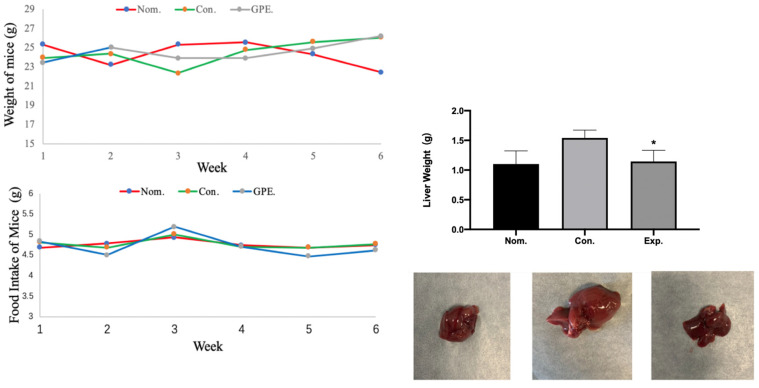
Body weight, food intake, liver weight and shape of liver in different group of mice (n = 6). (* *p* < 0.05).

Figure 2 is based on the record of daily food intake and body weight. It was found that, during the experiment time, there was no clear significant difference in food intake and body weight of mice among all three groups. In addition, hypertrophy was found in the livers of the GPE group despite no significant difference in liver weight.

### 2.2. GPE Alleviated HFCD-Induced Atherosclerotic Lesions

**Figure 3:**
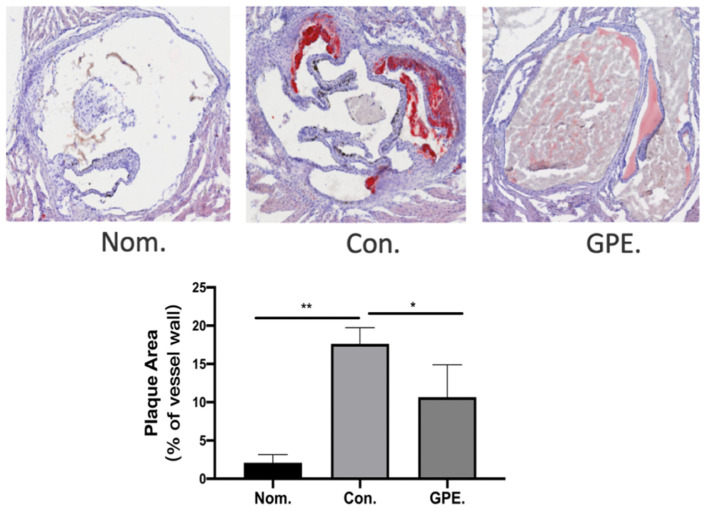
GPE alleviated HCD-induced atherosclerotic lesions (n = 6). (* *p* < 0.05, ** *p* < 0.01).

Mice hearts were cryo-sected to obtain the aortic root. Oil Red O was used to visualize atherosclerotic lesions of the aorta root in cross-section. The staining of the aortic sinus also showed much more severe atherosclerotic lesions in the control group. However, compared with the NOM group, atherosclerotic lesions were significantly reduced in the GPE group (Figure 3). These results showed that GPE could reduce HFCD-induced atherosclerotic lesions.

### 2.3. GPE Had Lipid-Lowering Effects

**Figure 4:**
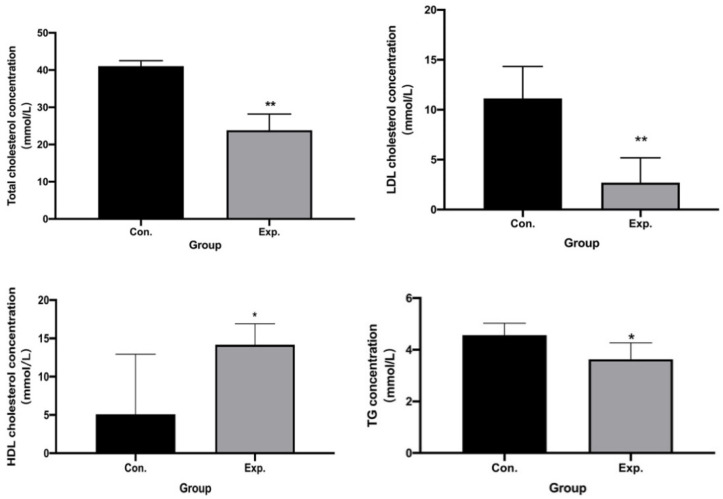
GPE had lipid-lowering effects in TC, TG, LDL-C, HDL-C level (n = 6). (* *p* < 0.05, ** *p* < 0.01).

Compared with the HFCD group, w can see the levels of TC, LDL-C, and TG of GPE group, while HDL-C level was increased in GPE group (*p* < 0.05) (Figure 4).

### 2.4. GPE Inhibited TMA/TMAO Metabolism

**Figure 5:**
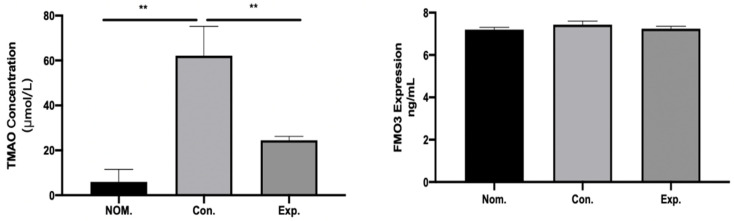
The concentrations of TMAO in plasma were reversed by GPE and FMO3 in all groups with no significant difference (n = 6). (** *p* < 0.01).

The differences in TMAO metabolism in mice treated with or without GPE were evaluated. The concentrations of TMAO in plasma in the CON group were much higher than those in the NOM group (*p* < 0.01), which means that HFCD can induce a high level of TAMO in the serum of model mice. After treatment with GPE, the concentrations of TMAO in plasma (*p* < 0.01) were reversed, which indicate that GPE can reduce the level of TMAO. On the other hand, the concentrations of FMO3 in all groups showed no significant difference (Figure 5), indicating that the changes of TMAO level in plasma would not be influenced by metabolism, and only influenced by the production of TMA by gut microbiota.

### 2.5. Effect of GPE Treatment on Gut Microbiota in ApoE^−/−^ Mice

**Figure 6:**
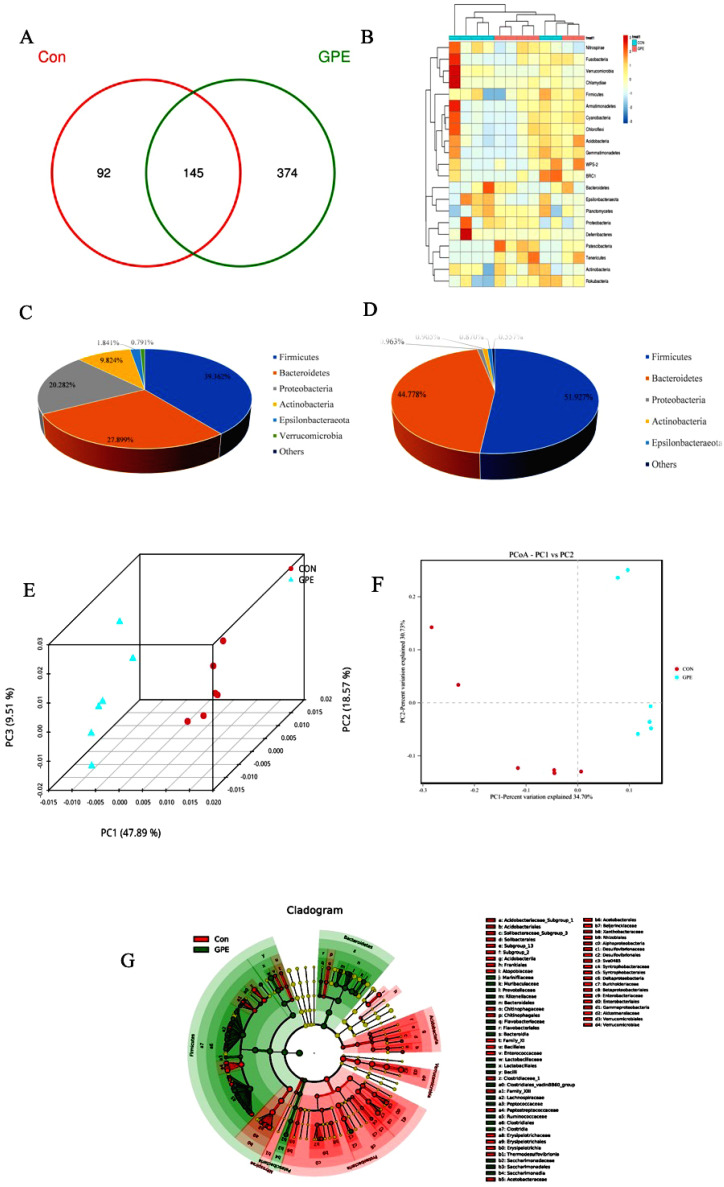
Effect of GPE treatment on gut microbiota in ApoE^−/−^ mice: Venn diagram analysis of unique/shared OTUs in the gut microbiota (**A**); Microbial community heatmap analysis (**B**); Major variations of the structure of the gut microbiota of mice without (**C**) or with (**D**) GPE treatment; PCA analysis (**F**) and uniFrac-based PCOA analysis (**E**); LEfSE analysis (**G**), respectively.

It was hypothesized that GPE alleviated atherosclerotic lesions and postponed the degeneration of atherosclerosis through the gut microbiota. Illumina MiSeq 16S rRNA amplicon sequencing was conducted, and Chao index, Simpson index, and Shannon index revealed that GPE treatment significantly increased the abundance of gut microbiota (Table 1). PCoA demonstrated significant variation in microbiota composition between the CON group and the GPE group. The Venn diagrams also showed the significant changes in compositional similarity and overlaps between different groups in the OTU level. It was found that the main intestinal microbiota of the HFCD fed CON group were Firmicutes (39.362%), Verrucomicrobia (27.899%), Bacteroidetes (20.282%), Proteobacteria (9.824%), and Actinobacteria (1.841%). The main intestinal microflora of the GPE group were different from CON: Firmicutes (51.927%), Bacteroidetes (44.778%), Proteobacteria (0.963%), and Actinobacteria (0.905%). Though the Verrucomicrobia was relatively highly abundant in the CON group, it declined significantly in abundance in the GPE group and could not be called a major bacterium (Figure 6).

**Figure 7:**
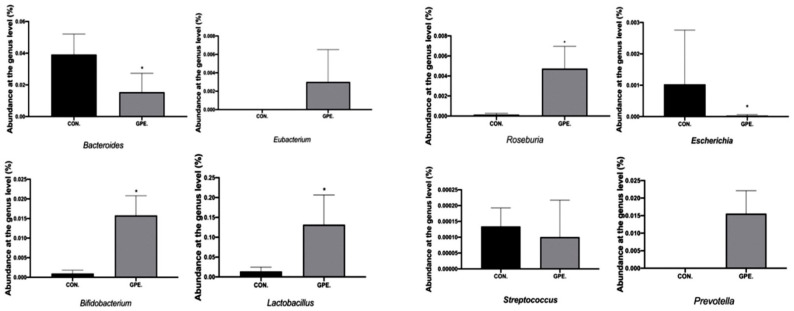
Analysis of bacterial species composition at the genus level. (* *p* < 0.05).

In addition, the abundance of Bacteroidetes and Firmicutes were paid attention to, as these bacteria account for the majority in the intestinal community of healthy people. The Firmicutes/Bacteroidetes ratio in the gut microbiota is an important health indicator, and the this increased significantly after treatment with GPE. The relative abundance of trimethylamine-producing intestinal bacteria decreased, such as *Clostridioides and Desulfovibrionaceae,* while the relative abundance of other trimethylamine-producing bacteria such as *Klebsiella Pneumoniae* and *Proteus Vulgaris* did not change significantly. It was also found that bacteria which can product butyrate, such as *Eubacterium, Roseburia, Bifidobacterium, Lactobacillus, and Prevotella,* increased significantly after treatment with GPE (Figure 7).

### 2.6. In Vivo Antioxidant Capacity

**Figure 8:**
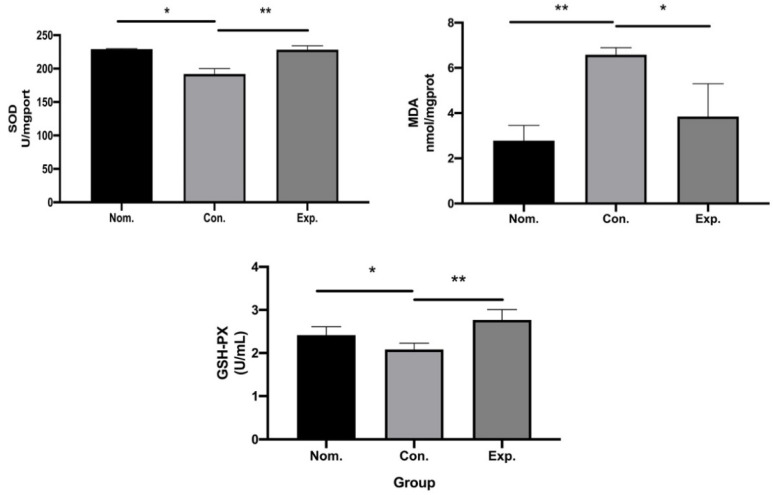
The antioxidant capacity of GPE in normal group, control group and GPE group (n = 6), respectively in vivo. (* *p* < 0.05, ** *p* < 0.01).

The detection of oxidative stress-related enzymes in HFCD-induced atherosclerosis mice showed that activities of GSH-Px and SOD were lower in the GPE-treated group while the concentration of MDA was higher in the HFCD-induced atherosclerosis CON group compared with the NOM group. Meanwhile, compared with the HFCD-induced atherosclerosis CON group, activities of SOD and GSH-Px increased, and the concentration of MDA levels decreased when treated with GPE (Figure 8).

### 2.7. GPE Supplementation Reduces Inflammation in the Liver

**Figure 9:**
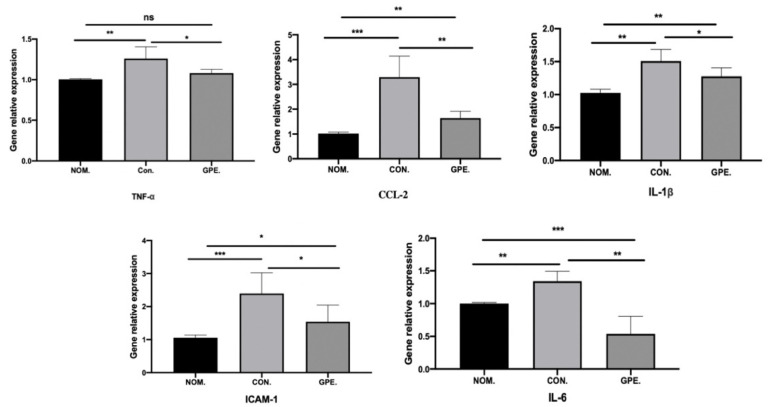
The relative gene expression of IL-6, TNF-α, IL-1β, CcL-2, ICAM-1 in mice (n = 6). (* *p* < 0.05, ** *p* < 0.01 and *** *p* < 0.001).

The TNF-α, IL-6, and IL-1β mRNA expression levels were measured in the HFCD-induced atherosclerosis CON group compared with those of the NOM group, while the mRNA expression level of IL-6, IL-1β, and TNF-α decreased in the GPE treated group (*p* < 0.05), and some of the inflammatory cytokines reached the average level. Moreover, ICAM-1 and Ccl2 mRNA expression was elevated in the HFCD-induced atherosclerosis CON group compared with the NOM group, while the mRNA expression of those in the HFCD-induced atherosclerosis with GPE treated group decreased significantly (*p* < 0.05) (Figure 9).

### 2.8. Intestinal SCFA Metabolites

**Figure 10:**
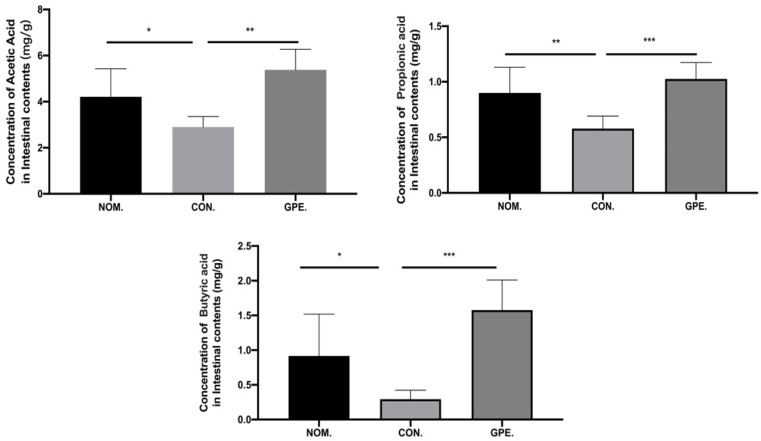
SCFAs concentrations of the fecal sample in different groups. (* *p* < 0.05, ** *p* < 0.01, and *** *p* < 0.001).

We examined the effect of GPE on core metabolite and SCFA production by gut microbes. Compared with the model group, the GPE group demonstrated higher SCFA concentrations in the fecal sample, such as Acetic Acid (2.896 mg/g vs. 5.381 mg/g, *p* < 0.01), Propionic Acid (0.579 mg/g vs. 1.025 mg/g, *p*<0.01), and Butyric Acid (0.293 mg/g vs. 1.578 mg/g, *p* < 0.01) (Figure 10).

### 2.9. Significant Intestinal Metabolic Changes during GPE Intervention

After 6 weeks of treatment, the incidence of atherosclerotic lesions in the arteries and aortic sinuses of each group of mice was assessed. In the CON group of ApoE^−/−^ mice, atherosclerotic plaques in the aortic sinus were all significantly increased after 6 weeks of HCD treatment, whereas GPE treatment inhibited the progression of atherosclerosis. Meanwhile, the untargeted and targeted metabolomics based on GC-MS were used to obtain intestinal metabolic profiles and to identify regulators associated with the anti-atherogenic effects of GPE.

The PCA score plot indicated that the intestinal metabolic profiles of the normal feed group were significantly different from those of the HFCD-induced atherosclerosis CON group, and there were significant differences in the metabolic profiles of the CON and GPE groups. Then, 63 detected metabolites were identified as differential metabolites associated with the formation of atherosclerosis and effect of GPE (Table 2). This showed substantial changes in intestinal metabolites involved in amino acid metabolism, carbohydrate metabolism, tricarboxylic acid cycle, lipid metabolism and other metabolic pathways during GPE treatment. Further pathway analysis revealed that 29 metabolic pathways were disturbed to varying degrees during GPE treatment, particularly the citric acid cycle (TCA cycle), fatty acid biosynthesis, galactose and glycero-lipid-metabolism biosynthesis of unsaturated fatty acids.

## 3. Discussion

Knowledge of the role of microbes in human health and disease is growing. The different compounds influencing gut microbes probably play an important role in human health. Although research in “the microbiome as drug” is still in its infancy, drug discovery in the field of the microbiome has enormous untapped potential. The results suggest that the possible reason for GPE attenuating atherosclerotic lesions significantly and delaying the progression of atherosclerosis is the effect of GPE on the gut microbiota of ApoE^−/−^ mice. As a key factor in atherosclerosis, the maladaptive inflammatory response to subcortical lipoproteins leads to the failure of inflammation resolution, promoting the transformation of the atherosclerotic lesion into a more severe plaque [14].

Relevant studies have shown that there is dyslipidemia in atherosclerosis which is characterized by increased LDL-C and TG and decreased HDL-C, indicating that atherosclerotic mice had higher TG, TC and LDL-C levels and lower HDL-C levels [15]. Increased serum LDL-C levels are widely recognized as an important manifestation that promotes the risk of endothelial dysfunction and the progression of atherosclerosis [16]. After gavage treatment of mice with GPE, the levels of the above indicators were reversed to varying degrees. The increased TC and LDL-C contributed to the development of atherosclerosis. Moreover, the low level of HDL-C is correlated with an enhanced risk of cardiovascular disease.

According to reports, the oxidative stress caused by disruption of the balance between antioxidants and reactive oxygen radicals, which included excessive production of reactive oxygen species and reduction of antioxidant activity in the body, has been implicated in the progression of several CVD, including atherosclerosis [17]. Oxidative damage in HFCD-induced ApoE^−/−^ mice decreased after GPE treatment, with increased SOD and GSH-Px, and decreased MDA. The two antioxidant enzymes with high efficiency, SOD and GSH-Px, could convert highly reactive free radicals into less reactive ones. Thus, the formation of water and molecular oxygen or the conjugation with harmful compounds was prevented, and safer substances were produced instead of toxic [18]. In particular, this can prevent lipid peroxidation and reduce the risk of atherosclerosis.

In atherosclerosis, elevated expression of ICAM-1, VCAM-1, and selectins are essential markers of endothelial dysfunction. ICAM-1 is involved in atherosclerosis development by regulating the adhesion of inflammatory cells such as monocytes to endothelial cells and their entry into the sub-endothelium. Several stimuli such as LDL, TNF-α, and high glucose can upregulate ICAM-1 expression in endothelial cells and increase monocyte-endothelial cell adhesion [19]. In addition, it has been proved that inflammation drives the formation, development and rupture of atherosclerotic plaques, and the proinflammatory factors are produced by the inflammatory subset of monocytes or the accumulation of macrophages [20]. In this study, atherosclerotic plaque area of aorta in GPE group decreased. Furthermore, the expression of inflammatory cytokines TNF-α and IL-6 were decreased. The increased inflammation cytokines, including IL-6, IL-1β, and TNF-α, are also the main characteristics of atherosclerosis [21].

*Staphylococcus*, *Klebsiella pneumoniae*, *Streptococcus*, and *Proteus vulgaris* have been found in both atherosclerotic lesions and intestinal tract of the same individual. This finding suggests that the intestinal microbiota is involved in the formation and development of atherosclerosis [22]. It has been found that atherosclerotic patients show a relative reduction in *Prevotella* and *Bacteroides,* but increasing in *Escherichia* and *Streptococcus* [23]. Similar findings in this study had an average enrichment of *Prevotella* in the gut when atherosclerosis symptoms began to resolve and a decrease of *Streptococcus* and *Escherichia*. However, patients in Poland with abnormal cholesterol concentration and LDL-C levels have rich *Prevotella*, low *Clostridium* and *Faecalibacterium* proportion [24]. The relative depletion of butyrate producing bacteria (*Roseburia* and *Eubacteria*) was negatively correlated with the development of atherosclerotic lesions in patients and genetic mouse models [25]. Therefore, the changes in the various butyrate-producing bacteria were investigated. The relative consumption of *Roseburia* and *Eubacterium* can produce butyrate, which is inversely correlated with the development of atherosclerotic lesions in patients and genetic mice models [26]. Previous studies have shown that the increase in TMAO levels may be related to the increase in the proportion of Firmicutes or the decrease of Bacteroides proportion [27]. However, it is not clear which species play a dominant role in the formation of atherosclerosis [28]. The mechanism of how the gut microbiota affects the development of atherosclerosis is an important topic for future research.

In depth analysis of the gut microbiota revealed that trimethylamine lyase secreted by gut bacteria was affected, thereby affecting the process of choline conversion to TMA, resulting in a decrease in TMAO levels. According to non-targeted metabolomics analysis, metabolic pathways were significantly disturbed, including citrate cycle (TCA cycle), galactose and glycerolipid metabolism biosynthesis of unsaturated fatty acids; fatty acid biosynthesis. TCA is the center of carbohydrate, protein and lipid metabolism in the body. In particular, acetyl-CoA from citric acid, an intermediate product of the TCA cycle, are essential for lipid biosynthesis, which contributes significantly to lipid storage in mammals [29]. Thus, certain effects on lipid metabolism occur when there are changes in TCA-related enzymes. Choline-TMA-TMAO metabolic pathway interacts with lipid metabolism; changes in body lipid metabolism can affect the choline-TMA-TMAO pathway, while choline metabolism, based on gut microbiota, can influence metabolic function in many ways. [30,31]. How much of the gut metabolic pathways change affect the development of atherosclerosis is also a major topic for future research.

Through this animal experimental study, we found that gypenoside XLIX can alleviate atherosclerosis by regulating the intestinal microbiota, inhibiting trimethylamine production, alleviating inflammatory response and restraining oxidative stress. However, whether it is effective in clinical practice is worth further study. In the future, we will study the anti-atherosclerosis effect of gypenoside XLIX in combination with clinical practice.

## 4. Materials and Methods

### 4.1. Reagents

Gypenoside XLIX (GPE) was purchased from Wuhan Weiss Biological Engineering Co., Ltd. (Wuhan, China) (purity >98%). Choline was purchased from Shanghai Macklin Biochemical Technology Co., Ltd. (Shanghai, China).

### 4.2. Ethics Statement

In this research, the use of mice was approved by the China Pharmaceutical University Animal Care and Use Committee (20210412) and all experimental animal procedures followed the guidelines of the Institute Animal Care and Use Committee of China Pharmaceutical University.

### 4.3. Animal and Choline Treatment

After one-week acclimatization, 24 ApoE^−/−^ mice were divided into 3 groups randomly: NOM group (normal feed group: regular diet, physiological saline by gavage), CON group (HFCD diet, physiological saline by gavage), and GPE group (HFCD diet with 30 mg kg^−1^ d^−1^ GPE by gavage). The regular commercial mice feeds contained 6.2% fat, 35.6% carbohydrate, 20.8% protein, and the calorific value: 17.6 KJ/g. The HFCD mice feeds contained 0.15% cholesterol, 21% fat, and 1% choline chloride. The NOM group was set up to observe the physiological indexes of normal mice. Changing the water and treatments were processed daily for 6 weeks.

In the end, mice were anesthetized with 50 mg/kg by gavage secobarbital. Blood was collected from orbital veins, and serum were separated from whole blood by centrifugation (2500 rpm for 15 min at 4 °C) and stored at −80 °C immediately. Moreover, the heart tissues, liver tissues and whole aorta were taken.

### 4.4. Biochemical Analysis

The levels of triglyceride (TG) and total cholesterol (TC) in the liver, TG, TC, high-density lipoprotein (HDL-C), low-density lipoprotein (LDL-C) in serum were determined using commercial ELISA kits (Nanjing Jiancheng Bioengineering Institute, Nanjing, China) according to the manufacturer’s instructions.

### 4.5. Analysis of Atherosclerotic Lesions

After collecting blood samples, the circulatory system was flushed using a stroke-physiological saline solution. Heart and aortic tissue from the aortic root to the iliac bifurcation were taken and stored in 4% paraformaldehyde for 6 h, frozen in liquid nitrogen and cut into 5 mm sections, then subjected to Oil Red O staining to analyze atherosclerotic lesions. The percentage of atherosclerotic lesion area shown by oil red O staining represents the relative severity of atherosclerosis. The area and size of the lesions were quantified by Image J.

### 4.6. Detection of FMO3 and TMAO in Mice

Since FMO3 in the liver was important to TMA/TMAO cycling, the protein expression of FMO3 was detected by an enzyme-linked immunosorbent assay according to the manufacturer’s protocol (Shanghai Jining Shiye, Shanghai, China). Briefly, mouse liver was homogenized (1.0 g of liver sample in 10 mL ice cold 0.9% NaCl solution), and then centrifuged at 3000 rpm for 10 min at 4 °C, and the supernatant was used to determine the content of FMO3. The concentrations of TMAO in plasma were then assessed by the ELISA method according to the manufacturer’s protocol (Shanghai Jining Shiye, Shanghai, China).

### 4.7. The Effect of GPE on Gut Microbiota

The V3-V4 region of the bacterial 16S rRNA gene was amplified with extracted DNA as a template using the Applied Biosystems PCR system (GeneAmp^®^ 9700, ABI, Foster City, CA, USA). The amplified products were then purified by TIANGEN DNA gel purification kit (TIANGEN Mini Purification Kit, TIANGEN, Beijing, China). MiSeq library construction and sequencing were performed and bioinformation analyzed as described by Segata et al. [32].

### 4.8. In Vivo Antioxidant Capacity of GPE

Glutathione peroxidase (GSH-Px) in the liver, malondialdehyde (MDA), and superoxide dismutase (SOD) in serum were determined with the commercial test kits (Nanjing Jiancheng Institute of Biological Engineering) following the manufacturer’s instructions to show the effect of antioxidants in vivo.

### 4.9. RNA Isolation and Real-Time PCR

Total RNA was extracted from liver tissues of the NOM, CON and GPE groups using Trizol reagent (Thermo Fisher, Waltham, MA, USA). The concentration and integrity of extracted RNA were detected by Nanodrop 2000 and PCR analyze, respectively. The RNA was reverse transcribed to cDNA using PrimeScript™ RT reagent Kit (TaKaRa, Japan). ABI 7500 Real-Time PCR System (Applied Biosystems, Foster City, CA, USA) was used to conduct Real-time PCR. The relative expression of every target gene was normalized and the relative expression data were analyzed using the 2^−ΔΔCt^ method. The primer sequences of detected genes are listed in Table 3.

### 4.10. SCFA Analysis by HSGC/MS

SCFA analysis was accomplished by Agilent 7890 gas chromatograph (Agilent Corporation, Santa Clara, CA, USA) coupled with a 5975c inert MSD quadrupole mass spectrometer, and a DB-FFAP capillary column (30 mm × 0.25 mm i.d., 0.25 mm film thickness). 0.1 g of homogenized fecal sample was mixed with 1 mL of 6% H_3_PO_4_ solution and ultrasonic for 3 min, then placed in a headspace autosampler at 80 °C for 30 min. Subsequently, 1 mL samples were injected into the column at 80 °C. Helium gas was used as a carrier for the sample and passed through the column at a constant flow rate of 1 mL/min. The oven was preheated at 50 °C for 1 min, then heated up 10 °C/min to 200 °C. The temperature of ion source was 250 °C and temperature of syringe also was 250 °C. With ionization energy of 70 eV, the mass detector system was operated in EI mode. The monitored ion data were collected between m/z 33 to 200. Short chain fatty acids were quantified by the mass spectrometry Library of the National Institute of Standards and Technology.

### 4.11. GC-TOF-MS-Based Metabolomics Analyses

Based on the methods of Yang et al. [33] and Wen et al. [34], metabolomics analyses were performed by gas chromatography. DB-5MS capillary column (30 m × 0.25 mm i.d., 0.25 μm film thickness, Agilent J & W Scientific, Folsom, CA, USA) was used to separate the derivatives.

### 4.12. Statistical Analysis

Differences between the two groups were assessed using the t-test, while differences between multiple groups were compared using one-way analysis of variance (ANOVA). Data were analyzed using the Statistical Package for the Social Sciences (SPSS) 26.0 (SPSS, Inc., Chicago, IL, USA) and GraphPad Prism 8 was used for graphics. *p* < 0.05 was considered to be a statistically significant difference.

## 5. Conclusions

*Gynostemma pentaphyllum* is frequently prescribed to treat cardiovascular diseases in China. In this study, we found that gypenoside XLIX can reduce atherosclerosis and the anti-atherosclerotic effect of GPE is related to the substantial changes in intestinal microbiota and anti-inflammatory activity. This can provide a certain theoretical basis for the treatment of atherosclerosis with Chinese herbal medicine.

## Figures and Tables

**Table 1:** α diversity analysis of gut microbiota in CON and GPE group.

	CON.	GPE.	*p* Value
Shannon	3.21 ± 0.02	4.39 ± 0.16	0.01
Simpson	0.12 ± 0.03	0.03 ± 0.01	0.04
Chao	204.30 ± 7.72	451.03 ± 10.13	0.01
Coverage	0.99 ± 0.00	0.99 ± 0.00	

**Table 2:** Differential metabolites related to atherosclerosis in GPE treated HFCD diet group compared with HFCD diet.

Metabolite	Fold Change
D-Cellobiose	22.449
Tyramine	13.37
Melibiose	13.235
Dihydro-cholesterol	12.633
5-Methoxytryptamine	10.771
4-O-Hexopyranosylhex-2-ulofuranose	9.1412
D-Galacturonic cid	8.8722
Hydroxyproline	7.9615
3-Methyl-2-oxobutanoic acid	5.8605
Hexose	5.4677
3-Methyl-2-oxovalerate	5.4058
Oxalacetic acid	5.0682
L-Phenylalanine	3.7558
3-Methyl-2-oxopentanoate	3.7309
4-Methyl-2-oxopentanoate	3.6265
Malbit	3.5755
Deoxyadenosine	3.5743
Galactosyl-glycerol	3.5642
L-Gulcono-1,4-lactone	3.2961
N-Methyl-DL-alanine	3.0154
D-Lyxose	2.9811
L-Iditol	2.9403
2-O-Methyl-D-mannopyranosa	2.5005
Pentose	2.4453
Deoxyinosine	2.323
Pantothenic acid	2.1301
Pseudo-uridine	2.0331
Thymine	1.9163
D-Tagatose	1.8483
D-Glucose	1.7846
2,5,7,8-Tetramethyl-2-(5,9,13-trimethyltetradecyl)-3,4-dihydro-2h-chromen-6-ol	1.7703
Linoleate	1.7462
Aldohexose	1.5223
Aminomalonate	1.2711
Di-isopropanolamine	1.268
Glycerol	0.6751
Hexa-decanoic acid	0.6641
Propane-1,3-diol	0.66263
Lactic acid	0.66201
gamma-Aminobutyric acid	0.59821
L-Alanyl-L-alanine	0.53075
Glycolic acid	0.52183
Octadecanoic acid	0.51757
Malic acid	0.46053
Chimyl alcohol	0.41685
4-Hydroxylphenyllactic acid	0.38248
Cholestanol	0.3759
Batyl alcohol	0.37026
2,3-Bisphospho-glyceric acid	0.29243
Creatine	0.28688
2,3-Diaminopropionic acid	0.26765
3-Epicholic acid	0.19065
Icosanoic acid	0.18807
11beta-Hydroxyandrostenedione	0.15355
4-Hydroxyphenylacetic acid	0.14495
1-Eicosanol	0.13899
Butenedioic acid	0.11864
2-Deoxy-D-galactose	0.11572
L-Aspartate	0.097594
Octadecanol	0.070298
3,4-Dihydroxyphenylacetic acid	0.028664
Cholan-24-oic acid, 3,7,12-trihydroxy-, (3alpha,5beta,7alpha,12alpha)-	0.0058382
Cholic acid	0.0040922

**Table 3:** PCR gene specific primer pairs.

Gene	Primer Sequence (5′-3′)
IL-1β	FOR: GGTCAAAGGTTTGGAAGCAG REV: TGTGAAATGCCACCTTTTGA
IL-6	FOR: AGGGTCTGGGCCATAGAACT REV: CCACCACGCTCTTCTGTCTAC
TNF-α	FOR: AGGGTCTGGGCCATAGAACT REV: CCACCACGCTCTTCTGTCTAC
ICAM-1	FOR: AACAGTTCACCTGCACGGAC REV: GTCACCGTTGTGATCCCTG
Ccl2	FOR: ATTGGGATCATCTTGCTGGT REV: CCTGCTGTTCACAGTTGCC
β-actin	FOR: GCTGTGCTATGTTGCTCTAG REV: CGCTCGTTGCCAATAGTG

## Data Availability

The raw data for 16S rRNA gene sequence have been deposited in the NCBI BioProject database (https://www.ncbi.nlm.nih.gov/bioproject/, 10 February 2022) under accession number: PRJNA769542. The other data of this study are available on request from W.C.
